# Supplementary material for: Genetic relatedness of serial rectal isolates of Acinetobacter baumannii in an adult intensive care unit of a tertiary hospital in Kuwait
Source: PLoS One. 2020 Apr 2;15(4):e0230976. doi: 10.1371/journal.pone.0230976 (PMC7127897; doi:10.1371/journal.pone.0230976)
Supplement: S1 Table — (DOCX) [file pone.0230976.s001.docx]

**S1 Table. The relationship of colonies by DiversiLab dendrograms among similar and different morphotypes.**

| **Serial no. of patients** | **No. of colony types** | **No. of colonies studied** | **Relationship of colonies by DiversiLab dendrogram** | | |
| --- | --- | --- | --- | --- | --- |
|  |  |  | I | R | U |
| **1** | 1 | 3 | 3 | 0 | 0 |
| **2** | 2 | 2 | 0 | 0 | 2 |
| **3** | 3 | 3 | 0 | 0 | 3 |
| **4** | 3 | 3 | 0 | 2 | 1 |
| **5** | 1 | 3 | 3 | 0 | 0 |
| **6** | 4 | 4 | 0 | 2 | 2 |
| **7** | 1 | 3 | 3 | 0 | 0 |
| **8** | 6 | 6 | 0 | 2 | 4 |
| **9** | 1 | 3 | 3 | 0 | 0 |
| **10** | 1 | 3 | 3 | 0 | 0 |
| **11** | 3 | 3 | 0 | 3 | 0 |
| **12** | 2 | 2 | 0 | 0 | 2 |

I=identical, R=related, U=unrelated
