# Supplementary material for: Genetic relatedness of serial rectal isolates of Acinetobacter baumannii in an adult intensive care unit of a tertiary hospital in Kuwait
Source: PLoS One. 2020 Apr 2;15(4):e0230976. doi: 10.1371/journal.pone.0230976 (PMC7127897; doi:10.1371/journal.pone.0230976)
Supplement: S2 Table — (DOCX) [file pone.0230976.s002.docx]

Table S2. Antimicrobial susceptibilities of 108 serial rectal *A. baumannii* isolates from 13 patients.

| **Patient isolates** | **MIC (resistance or susceptibility) to** | | | | | | | | | | | | | | | |
| --- | --- | --- | --- | --- | --- | --- | --- | --- | --- | --- | --- | --- | --- | --- | --- | --- |
|  | **Amikacin** | **Cefepime** | **Cefotaxime** | **Ceftazidime** | **Ceftriaxone** | **Ciprofloxacin** | **Colistin** | **Gentamicin** | **Imipenem** | **Levofloxacin** | **Meropenem** | **Piperacillin** | **Piperacillin /tazobactam** | **Tetracycline** | **Tigecycline** | **Trimethoprim/ sulfamethoxazole** |
| **A1** | 6(S) | 256(R) | 256(R) | 4(S) | 32(R) | 32(R) | 0.125(S) | 1024 (R) | 32 (R) | 4(I) | 32 (R) | 256(R) | 256(R) | 256(R) | 2(R) | 1(S) |
| **A2** | 4(S) | 96(R) | 256(R) | 1.5(S) | 24(I) | 32(R) | 0.125(S) | 1024 (R) | 32 (R) | 4(I) | 32 (R) | 256(R) | 256(R) | 256(R) | 2(R) | 4(R) |
| **A3** | 256(R) | 256(R) | 256(R) | 256(R) | 256(R) | 32(R) | 0.125(S) | 1024 (R) | 32 (R) | 16(R) | 32 (R) | 256(R) | 256(R) | 256(R) | 3(R) | 32 (R) |
| **A4** | 256(R) | 64(R) | 256(R) | 256(R) | 256(R) | 32(R) | 0.75(S) | 1024 (R) | 32 (R) | 8(R) | 32 (R) | 256(R) | 256(R) | 256(R) | 3(R) | 0.25(S) |
| **A5a** | 6(S) | 48(R) | 256(R) | 2(S) | 12(I) | 32(R) | 0.19(S) | 1024 (R) | 32 (R) | 8(R) | 32 (R) | 256(R) | 256(R) | 256(R) | 3(R) | 3(I) |
| **A5b** | 256(R) | 256(R) | 256(R) | 256(R) | 256(R) | 32(R) | 0.5(S) | 1024 (R) | 32 (R) | 12(R) | 32 (R) | 256(R) | 256(R) | 256(R) | 4 (R) | 32 (R) |
| **B1** | 256 (R) | 256 (R) | 256 (R) | 256 (R) | 256 (R) | 32(R) | 0.38(S) | 1024 (R) | 32 (R) | 12 (R) | 32 (R) | 256 (R) | 256 (R) | 256 (R) | 4 (R) | 32 (R) |
| **B2** | 256 (R) | 256 (R) | 256 (R) | 256 (R) | 256 (R) | 32(R) | 0.38(S) | 1024 (R) | 32 (R) | 12 (R) | 32 (R) | 256 (R) | 256 (R) | 256 (R) | 6 (R) | 1(S) |
| **B3** | 256 (R) | 256 (R) | 256 (R) | 256 (R) | 256 (R) | 32(R) | 0.25(S) | 1024 (R) | 32 (R) | 8 (R) | 32 (R) | 256 (R) | 256 (R) | 256 (R) | 6 (R) | 0.3(S) |
| **B4** | 256 (R) | 256 (R) | 256 (R) | 256 (R) | 256 (R) | 32(R) | 0.38(S) | 1024 (R) | 32 (R) | 8 (R) | 32 (R) | 256 (R) | 256 (R) | 256 (R) | 6 (R) | 32 (R) |
| **B5** | 256 (R) | 256 (R) | 256 (R) | 256 (R) | 256 (R) | 32(R) | 0.38(S) | 1024 (R) | 32 (R) | 12 (R) | 32 (R) | 256 (R) | 256 (R) | 256 (R) | 6 (R) | 32 (R) |
| **B6** | 256 (R) | 256 (R) | 256 (R) | 256 (R) | 256 (R) | 32(R) | 0.38(S) | 1024 (R) | 32 (R) | 0.38 (S) | 32 (R) | 256 (R) | 256 (R) | 256 (R) | 4 (R) | 32 (R) |
| **B7** | 256 (R) | 64 (R) | 256 (R) | 256 (R) | 256 (R) | 32(R) | 0.5 (S) | 1024 (R) | 32 (R) | 12 (R) | 32 (R) | 256 (R) | 256 (R) | 256 (R) | 4 (R) | 32 (R) |
| **G1a** | 256(R) | 256(R) | 256(R) | 256(R) | 256(R) | 32(R) | 0.38(S) | 1024 (R) | 32 (R) | 6(I) | 32 (R) | 256(R) | 256(R) | 256(R) | 2(R) | 0.5(S) |
| **G1b** | 256(R) | 256(R) | 256(R) | 256(R) | 256(R) | 32(R) | 0.5(S) | 1024 (R) | 32 (R) | 6(I) | 32 (R) | 256(R) | 256(R) | 256(R) | 4(R) | 0.5(S) |
| **G2** | 256(R) | 96(R) | 256(R) | 256(R) | 256(R) | 32(R) | 0.25(S) | 1024 (R) | 32 (R) | 16(R) | 32 (R) | 256(R) | 256(R) | 256(R) | 3(R) | 1(S) |
| **G3a** | 256(R) | 256(R) | 256(R) | 256(R) | 256(R) | 32(R) | 0.19(S) | 1024 (R) | 32 (R) | 32 (R) | 32 (R) | 256(R) | 256(R) | 256(R) | 4(R) | 1(S) |
| **G3b** | 256(R) | 256(R) | 256(R) | 256(R) | 256(R) | 32(R) | 0.5(S) | 1024 (R) | 32 (R) | 12(R) | 32 (R) | 256(R) | 256(R) | 256(R) | 3(R) | 0.75 (S) |
| **G4** | 256(R) | 64(R) | 256(R) | 256(R) | 256(R) | 32(R) | 0.75(S) | 1024 (R) | 32 (R) | 8(R) | 32 (R) | 256(R) | 256(R) | 256(R) | 3(R) | 0.5(S) |
| **G5a** | 256(R) | 48(R) | 256(R) | 256(R) | 256(R) | 32(R) | 0.25(S) | 1024 (R) | 32 (R) | 6(I) | 32 (R) | 256(R) | 256(R) | 256(R) | 2(R) | 0.38(S) |
| **G5b** | 256(R) | 256(R) | 256(R) | 256(R) | 256(R) | 32(R) | 0.25(S) | 1024 (R) | 32 (R) | 8(R) | 32 (R) | 256(R) | 256(R) | 256(R) | 3(R) | 0.38(S) |
| **G5c** | 256(R) | 96(R) | 256(R) | 256(R) | 256(R) | 32(R) | 0.5(S) | 1024 (R) | 32 (R) | 8(R) | 32 (R) | 256(R) | 256(R) | 256(R) | 3(R) | 0.38(S) |
| **I1** | 12(S) | 4(S) | 256(R) | 256(R) | 32(I) | 0.75(S) | 6(R) | 8(I) | 0.75(S) | 0.25(S) | 0.38(S) | 256(R) | 256(R) | 0.5(S) | 3(R) | 0.25(S) |
| **I2a** | 12(S) | 8(S) | 256(R) | 256(R) | 12(I) | 0.75(S) | 4(R) | 8(I) | 0.75(S) | 0.25(S) | 0.5(S) | 256(R) | 256(R) | 0.5(S) | 3(R) | 0.25(S) |
| **I2b** | 8(S) | 48(R) | 256(R) | 48(R) | 256(R) | 32(R) | 0.75(S) | 4(S) | 32 (R) | 32 (R) | 32 (R) | 256(R) | 256(R) | 256(R) | 6(R) | 4(R) |
| **I3a** | 12(S) | 48(R) | 256(R) | 64(R) | 256(R) | 32(R) | 0.5(S) | 6(I) | 32 (R) | 32 (R) | 32 (R) | 256(R) | 256(R) | 256(R) | 8(R) | 6(R) |
| **I3b** | 8(S) | 64(R) | 256(R) | 64(R) | 256(R) | 32(R) | 1(S) | 6(I) | 32 (R) | 32 (R) | 32 (R) | 256(R) | 256(R) | 256(R) | 4(R) | 2(S) |
| **I4** | 256(R) | 128(R) | 256(R) | 256(R) | 256(R) | 32(R) | 0.38(S) | 192(R) | 32 (R) | 8(R) | 32 (R) | 256(R) | 256(R) | 256(R) | 256(R) | 0.75(S) |
| **I5** | 256(R) | 256(R) | 256(R) | 256(R) | 256(R) | 32(R) | 0.38(S) | 1024 (R) | 32 (R) | 32 (R) | 32 (R) | 256(R) | 256(R) | 256(R) | 256(R) | 1.5(S) |
| **I6** | 8(S) | 24(I) | 256(R) | 2(S) | 64(R) | 32(R) | 0.094(S) | 1024 (R) | 32 (R) | 6(I) | 32 (R) | 256(R) | 256(R) | 256(R) | 2(R) | 6(R) |
| **I7a** | 12(S) | 32(R) | 256(R) | 3(S) | 32(I) | 32(R) | 0.38(S) | 1024 (R) | 32 (R) | 12(R) | 32 (R) | 256(R) | 256(R) | 256(R) | 3(R) | 8(R) |
| **I7b** | 8(S) | 48(R) | 256(R) | 6(S) | 48(I) | 32(R) | 0.5(S) | 1024 (R) | 32 (R) | 16(R) | 32 (R) | 256(R) | 256(R) | 256(R) | 3(R) | 8(R) |
| **I8** | 256(R) | 96(R) | 256(R) | 256(R) | 256(R) | 32(R) | 0.75(S) | 1024 (R) | 32 (R) | 32 (R) | 32 (R) | 256(R) | 256(R) | 256(R) | 6(R) | 1.5(S) |
| **I9** | 6(S) | 48(R) | 256(R) | 3(S) | 64(R) | 32(R) | 0.38(S) | 1024 (R) | 32 (R) | 8(R) | 32 (R) | 256(R) | 256(R) | 256(R) | 2(R) | 6(R) |
| **J1** | 256(R) | 256(R) | 256(R) | 256(R) | 256(R) | 32(R) | 0.38(S) | 1024 (R) | 32 (R) | 32 (R) | 32 (R) | 256(R) | 256(R) | 256(R) | 4(R) | 0.75(S) |
| **J2** | 256(R) | 256(R) | 256(R) | 256(R) | 256(R) | 32(R) | 0.19(S) | 1024 (R) | 32(R) | 32 (R) | 32 (R) | 256(R) | 256(R) | 256(R) | 3(R) | 0.38(S) |
| **J3a** | 256(R) | 256(R) | 256(R) | 256(R) | 256(R) | 32(R) | 0.5(S) | 1024 (R) | 32 (R) | 24(R) | 32 (R) | 256(R) | 256(R) | 256(R) | 6(R) | 0.75(S) |
| **J3b** | 256(R) | 256(R) | 256(R) | 256(R) | 256(R) | 32(R) | 0.125(S) | 1024 (R) | 24(R) | 32 (R) | 32 (R) | 256(R) | 256(R) | 256(R) | 2(R) | 0.5(S) |
| **J4a** | 256(R) | 64(R) | 256(R) | 256(R) | 256(R) | 32(R) | 0.38(S) | 1024 (R) | 16(R) | 32 (R) | 32 (R) | 256(R) | 256(R) | 256(R) | 6(R) | 0.75(S) |
| **J4b** | 256(R) | 4(S) | 256(R) | 256(R) | 256(R) | 32(R) | 0.125(S) | 1024 (R) | 16(R) | 32 (R) | 32 (R) | 256(R) | 256(R) | 256(R) | 6(R) | 1.5(S) |
| **J5** | 2(S) | 3(S) | 24(I) | 4(S) | 12(I) | 0.19(S) | 0.125(S) | 0.5(S) | 0.19(S) | 0.19(S) | 0.38(S) | 16(S) | 4(S) | 2(S) | 0.5(S) | 0.125(S) |
| **J6** | 4(S) | 1(S) | 4(S) | 1.5(S) | 256(R) | 0.064(S) | 0.094(S) | 1.5(S) | 0.19(S) | 0.32(S) | 0.38(S) | 4(S) | 0.064(S) | 3(S) | 0.25(S) | 0.064(S) |
| **J7** | 4(S) | 0.75(S) | 4(S) | 1(S) | 3(S) | 0.125(S) | 0.064(S) | 0.75(S) | 0.125(S) | 0.064(S) | 0.25(S) | 4(S) | 256(R) | 2(S) | 0.125(S) | 0.064(S) |
| **J8** | 4(S) | 1(S) | 6(S) | 2(S) | 8(S) | 0.125(S) | 0.094(S) | 0.5(S) | 0.125(S) | 0.094(S) | 0.38(S) | 8(S) | 0.032(S) | 2(S) | 0.5(S) | 0.094(S) |
| **J9** | 6(S) | 6(S) | 256(R) | 6(S) | 48(I) | 0.5(S) | 0.38(S) | 1.5(S) | 0.125(S) | 0.38(S) | 0.75(S) | 32(I) | 12(S) | 256(R) | 8(R) | 0.5(S) |
| **J10a** | 4(S) | 6(S) | 256(R) | 4(S) | 64(R) | 0.5(S) | 0.5(S) | 1(S) | 0.125(S) | 0.5(S) | 0.75(S) | 24(I) | 8(S) | 256(R) | 4(R) | 0.38(S) |
| **J10b** | 4(S) | 2(S) | 256(R) | 4(S) | 64 (R) | 0.5(S) | 1(S) | 1.5(S) | 0.25(S) | 0.5(S) | 1.5(S) | 32(I) | 0.032(S) | 256(R) | 6(R) | 0.38(S) |
| **J11** | 3(S) | 6(S) | 256(R) | 6(S) | 48(I) | 0.75(S) | 0.38(S) | 0.5(S) | 0.25(S) | 0.5(S) | 1(S) | 24(I) | 32(I) | 256(R) | 4(R) | 0.19(S) |
| **J12a** | 256(R) | 64(R) | 256(R) | 2(S) | 96(R) | 6(R) | 3(I) | 64(R) | 2(S) | 4(I) | 0.19(S) | 0.5(S) | 0.5(S) | 64(R) | 4(R) | 0.016(S) |
| **J12b** | 3(S) | 6(S) | 256(R) | 6(S) | 16(I) | 0.75(S) | 0.38(S) | 0.75(S) | 0.19(S) | 0.5(S) | 0.75(S) | 24(I) | 16(S) | 256(R) | 8(R) | 0.125(S) |
| **K1** | 256 (R) | 256 (R) | 256 (R) | 256 (R) | 256 (R) | 32(R) | 1(S) | 1024 (R) | 32 (R) | 8(R) | 32 (R) | 256 (R) | 256 (R) | 256 (R) | 4(R) | 32 (R) |
| **K2** | 256 (R) | 256 (R) | 256 (R) | 256 (R) | 256 (R) | 32(R) | 1(S) | 1024 (R) | 32 (R) | 8(R) | 32 (R) | 256 (R) | 256 (R) | 256 (R) | 4(R) | 8(R) |
| **K3** | 256 (R) | 256 (R) | 256 (R) | 256 (R) | 256 (R) | 32(R) | 1(S) | 1024 (R) | 32 (R) | 12(R) | 32 (R) | 256 (R) | 256 (R) | 256 (R) | 4(R) | 12(R) |
| **K4** | 256 (R) | 256 (R) | 256 (R) | 256 (R) | 256 (R) | 32(R) | 0.25(S) | 1024 (R) | 32 (R) | 2(S) | 32 (R) | 256 (R) | 256 (R) | 256 (R) | 1(S) | 2(S) |
| **K5** | 8 (S) | 1(S) | 16(I) | 0.5(S) | 8(S) | 0.125(S) | 1(S) | 1(S) | 0.25(S) | 0.094(S) | 0.25(S) | 16(S) | 2(S) | 3(S) | 0.25(S) | 0.094(S) |
| **K6** | 6(S) | 96(R) | 256 (R) | 64(R) | 256 (R) | 32(R) | 0.38(S) | 8(I) | 32 (R) | 4( I) | 32 (R) | 256 (R) | 256 (R) | 256(R) | 2 (R) | 32 (R) |
| **N1** | 256(R) | 256(R) | 256(R) | 256(R) | 256(R) | 32(R) | 1(S) | 1024 (R) | 32 (R) | 32 (R) | 32 (R) | 256(R) | 256(R) | 256(R) | 4(R) | 32 (R) |
| **N2** | 256(R) | 256(R) | 256(R) | 256(R) | 256(R) | 32(R) | 0.75(S) | 1024 (R) | 32 (R) | 32 (R) | 32 (R) | 256(R) | 256(R) | 256(R) | 4(R) | 32 (R) |
| **N3** | 256(R) | 256(R) | 256(R) | 256(R) | 256(R) | 32(R) | 0.75(S) | 1024 (R) | 32 (R) | 32 (R) | 32 (R) | 256(R) | 256(R) | 256(R) | 6(R) | 32 (R) |
| **N4a** | 256(R) | 256(R) | 256(R) | 256(R) | 256(R) | 32(R) | 0.75(S) | 1024 (R) | 32 (R) | 32 (R) | 32 (R) | 256(R) | 256(R) | 256(R) | 4(R) | 32 (R) |
| **N4b** | 256(R) | 256(R) | 256(R) | 256(R) | 256(R) | 32(R) | 0.38(S) | 1024 (R) | 32 (R) | 32 (R) | 32 (R) | 256(R) | 256(R) | 256(R) | 6(R) | 32 (R) |
| **N5a** | 256(R) | 256(R) | 256(R) | 256(R) | 256(R) | 32(R) | 0.5(S) | 1024 (R) | 32 (R) | 32 (R) | 32 (R) | 256(R) | 256(R) | 256(R) | 6(R) | 32 (R) |
| **N5b** | 256(R) | 256(R) | 256(R) | 256(R) | 256(R) | 32(R) | 0.5(S) | 1024 (R) | 32 (R) | 32 (R) | 32 (R) | 256(R) | 256(R) | 256(R) | 4(R) | 32 (R) |
| **N6a** | 4(S) | 256(R) | 256(R) | 256(R) | 256(R) | 32(R) | 1(S) | 6(I) | 32 (R) | 32 (R) | 32 (R) | 256(R) | 256(R) | 256(R) | 6(R) | 32 (R) |
| **N6b** | 6(S) | 256(R) | 256(R) | 256(R) | 256(R) | 32(R) | 1(S) | 6(I) | 32 (R) | 32 (R) | 32 (R) | 256(R) | 256(R) | 256(R) | 6(R) | 32 (R) |
| **N7a** | 256(R) | 256(R) | 256(R) | 256(R) | 256(R) | 32(R) | 2(S) | 1024 (R) | 32 (R) | 32 (R) | 32 (R) | 256(R) | 256(R) | 256(R) | 4(R) | 32 (R) |
| **N7b** | 256(R) | 256(R) | 256(R) | 256(R) | 256(R) | 32(R) | 1(S) | 1024 (R) | 32 (R) | 32 (R) | 32 (R) | 256(R) | 256(R) | 256(R) | 6(R) | 32 (R) |
| **N8** | 256(R) | 256(R) | 256(R) | 96(R) | 256(R) | 32(R) | 0.125(S) | 1024 (R) | 32 (R) | 32 (R) | 32 (R) | 256(R) | 256(R) | 256(R) | 6(R) | 0.25(S) |
| **N9** | 256(R) | 256(R) | 256(R) | 256(R) | 256(R) | 32(R) | 0.38(S) | 1024 (R) | 32 (R) | 32 (R) | 32 (R) | 256(R) | 256(R) | 256(R) | 32(R) | 2(S) |
| **O1** | 16(S) | 0.75(S) | 3(S) | 0.75(S) | 12(I) | 0.19(S) | 1(S) | 4(S) | 0.25(S) | 0.094(S) | 0.5(S) | 4(S) | 0.32(S) | 6(I) | 0.75(S) | 0.25(S) |
| **O2** | 6(S) | 256 (R) | 256 (R) | 256 (R) | 256 (R) | 0.38(S) | 6 (R) | 1(S) | 0.75(S) | 0.75(S) | 0.5(S) | 256 (R) | 256 (R) | 32(R) | 3(R) | 0.19(S) |
| **O3** | 8(S) | 8(S) | 256 (R) | 6(S) | 96(R) | 0.75(S) | 3(I) | 2(S) | 0.19(S) | 0.5(S) | 0.75(S) | 48 (I) | 16 (S) | 12(I) | 2(R) | 0.19(S) |
| **O4** | 32(I) | 12(I) | 8(S) | 3(S) | 6(S) | 0.75(S) | 1(S) | 32(R) | 0.25(S) | 0.38(S) | 0.38(S) | 4(S) | 0.032(S) | 4(S) | 4(R) | 0.38(S) |
| **O5** | 256 (R) | 256 (R) | 256 (R) | 256 (R) | 256 (R) | 32(R) | 0.5(S) | 1024 (R) | 32 (R) | 6(I) | 32 (R) | 256 (R) | 256 (R) | 256(R) | 3(R) | 1(S) |
| **O6** | 256 (R) | 256 (R) | 256 (R) | 256 (R) | 256 (R) | 32(R) | 1(S) | 1024 (R) | 32 (R) | 12(R) | 32 (R) | 256 (R) | 256 (R) | 256(R) | 3(R) | 0.75(S) |
| **R1** | 4(S) | 256(R) | 256(R) | 256(R) | 256(R) | 32(R) | 1(S) | 6(I) | 32 (R) | 32 (R) | 32 (R) | 256(R) | 256(R) | 256(R) | 8(R) | 32 (R) |
| **R2** | 4(S) | 48(R) | 96(R) | 4(S) | 96(R) | 32(R) | 0.75(S) | 6(I) | 32 (R) | 32 (R) | 32 (R) | 256(R) | 256(R) | 256(R) | 3(R) | 12(R) |
| **R3a** | 6(S) | 96(R) | 48(I) | 4(S) | 96(R) | 32(R) | 1(S) | 6(I) | 32 (R) | 16(R) | 32 (R) | 256(R) | 256(R) | 256(R) | 3(R) | 6(R) |
| **R3b** | 8(S) | 256(R) | 256(R) | 32(R) | 256(R) | 32(R) | 2(S) | 16(R) | 32 (R) | 32 (R) | 32 (R) | 256(R) | 256(R) | 48(R) | 6(R) | 2(S) |
| **R4** | 4(S) | 256(R) | 256(R) | 24(I) | 256(R) | 32(R) | 0.75(S) | 16(R) | 32 (R) | 32 (R) | 32 (R) | 256(R) | 256(R) | 24(R) | 6(R) | 2(S) |
| **R5a** | 8(S) | 64(R) | 32(I) | 4(S) | 96(R) | 32(R) | 2(S) | 6(I) | 32 (R) | 12(R) | 32 (R) | 256(R) | 256(R) | 256(R) | 2(R) | 3(I) |
| **R5b** | 8(S) | 256(R) | 48(I) | 4(S) | 96(R) | 32(R) | 1(S) | 6(I) | 32 (R) | 24(R) | 32 (R) | 256(R) | 256(R) | 256(R) | 2(R) | 8(R) |
| **R6** | 6(S) | 256(R) | 64(R) | 4(S) | 96(R) | 32(R) | 0.25(S) | 4(S) | 32 (R) | 12(R) | 32 (R) | 256(R) | 256(R) | 256(R) | 3(R) | 6(R) |
| **S1** | 256 (R) | 96 (R) | 256 (R) | 256 (R) | 256 (R) | 32(R) | 0.75 (S) | 1024 (R) | 32 (R) | 6 (I) | 32 (R) | 256 (R) | 256 (R) | 256 (R) | 2 (R) | 0.38 (S) |
| **S2** | 256 (R) | 48 (R) | 256 (R) | 256 (R) | 256 (R) | 32(R) | 0.5 (S) | 1024 (R) | 32 (R) | 12 (R) | 32 (R) | 256 (R) | 256 (R) | 256 (R) | 3 (R) | 0.5 (S) |
| **S3a** | 256 (R) | 64 (R) | 256 (R) | 256 (R) | 256 (R) | 32(R) | 0.38 (S) | 1024 (R) | 24 (R) | 32 (R) | 32 (R) | 256 (R) | 256 (R) | 256 (R) | 2 (R) | 1 (S) |
| **S3b** | 256 (R) | 48 (R) | 256 (R) | 256 (R) | 256 (R) | 32(R) | 0.38 (S) | 1024 (R) | 24 (R) | 24 (R) | 24(R) | 256 (R) | 256 (R) | 256 (R) | 2 (R) | 1 (S) |
| **S4a** | 256 (R) | 64 (R) | 256 (R) | 256 (R) | 256 (R) | 32(R) | 0.5 (S) | 1024 (R) | 24 (R) | 24 (R) | 32 (R) | 256 (R) | 256 (R) | 256 (R) | 3 (R) | 1 (S) |
| **S4b** | 256 (R) | 96 (R) | 256 (R) | 256 (R) | 256 (R) | 32(R) | 0.38 (S) | 1024 (R) | 24 (R) | 32 (R) | 16(R) | 256 (R) | 256 (R) | 256 (R) | 3 (R) | 1 (S) |
| **S5** | 256 (R) | 48 (R) | 256 (R) | 256 (R) | 256 (R) | 32(R) | 0.125(S) | 1024 (R) | 24 (R) | 24 (R) | 32 (R) | 256 (R) | 256 (R) | 256 (R) | 4 (R) | 1 (S) |
| **S6** | 256 (R) | 64 (R) | 256 (R) | 256 (R) | 256 (R) | 32(R) | 0.125(S) | 1024 (R) | 16 (R) | 32 (R) | 32 (R) | 256 (R) | 256 (R) | 256 (R) | 2 (R) | 1 (S) |
| **V1** | 2 (S) | 256(R) | 256(R) | 256(R) | 256(R) | 16 (R) | 0.5 (S) | 24 (R) | 1 (S) | 4 (I) | 1 (S) | 256 (R) | 256(R) | 256 (R) | 3 (R) | 1.5 (S) |
| **V2** | 3( S) | 256(R) | 256(R) | 256(R) | 256(R) | 32(R) | 0.25 (S) | 24(R) | 1.5 (S) | 4 (I) | 1.5 (S) | 256(R) | 256(R) | 256 (R) | 5 (R) | 0.125 (S) |
| **V3** | 12 (S) | 64(R) | 256(R) | 256(R) | 256(R) | 32(R) | 0.38 (S) | 192(R) | 0.75 (S) | 6(I) | 1.5 (S) | 256(R) | 256(R) | 256(R) | 3(R) | 0.19 (S) |
| **V4** | 2 (S) | 256(R) | 256(R) | 256(R) | 256(R) | 32(R) | 0.38 (S) | 24(R) | 1 (S) | 4(I) | 1.5 (S) | 256(R) | 256(R) | 256(R) | 3(R) | 0.094 (S) |
| **V5** | 256 (R) | 256(R) | 256(R) | 256(R) | 256(R) | 32(R) | 0.38 (S) | 1024 (R) | 32 (R) | 8(R) | 32 (R) | 256(R) | 256(R) | 256(R) | 4(R) | 0.38 (S) |
| **Y1** | 256(R) | 48(R) | 256(R) | 126(R) | 256(R) | 32(R) | 0.19 (S) | 1024 (R) | 32 (R) | 8(R) | 32 (R) | 256(R) | 256(R) | 256(R) | 6(R) | 0.75(S) |
| **Y2** | 16(S) | 64(R) | 256(R) | 8(S) | 128(R) | 32(R) | 0.38(S) | 64(R) | 32 (R) | 24(R) | 32 (R) | 256(R) | 256(R) | 16(R) | 4(R) | 4(R) |
| **Y3** | 256(R) | 64(R) | 256(R) | 256(R) | 256(R) | 32(R) | 0.25(S) | 1024 (R) | 32 (R) | 32 (R) | 32 (R) | 256(R) | 256(R) | 256(R) | 12(R) | 32 (R) |
| **Y4** | 256(R) | 256(R) | 256(R) | 256(R) | 256(R) | 32(R) | 0.5(S) | 1024 (R) | 32 (R) | 16(R) | 32 (R) | 256(R) | 256(R) | 256(R) | 6(R) | 1.5(S) |
| **Y5a** | 256(R) | 256(R) | 256(R) | 256(R) | 256(R) | 32(R) | 0.38(S) | 1024 (R) | 32 (R) | 12(R) | 32 (R) | 256(R) | 256(R) | 256(R) | 6(R) | 0.75(S) |
| **Y5b** | 256(R) | 256(R) | 256(R) | 256(R) | 256(R) | 32(R) | 0.38(S) | 1024 (R) | 32 (R) | 32 (R) | 32 (R) | 256(R) | 256(R) | 256(R) | 12(R) | 2(S) |
| **AF1** | 256 (R) | 96 (R) | 256 (R) | 256 (R) | 256 (R) | 32(R) | 0.19 (S) | 1024 (R) | 16 (R) | 6 (I) | 32 (R) | 256 (R) | 256 (R) | 256 (R) | 1.5 (R) | 0.5 (S) |
| **AF2** | 256 (R) | 96 (R) | 256 (R) | 256 (R) | 256 (R) | 32(R) | 0.19 (S) | 1024 (R) | 16 (R) | 16 (R) | 16 (R) | 256 (R) | 256 (R) | 256 (R) | 8 (R) | 0.38 (S) |
| **AF3** | 256 (R) | 96 (R) | 256 (R) | 256 (R) | 256 (R) | 32(R) | 0.19 (S) | 1024 (R) | 32 (R) | 8 (R) | 32 (R) | 256 (R) | 256 (R) | 256 (R) | 3 (R) | 0.5 (S) |
| **AF4** | 256 (R) | 256 (R) | 256 (R) | 256 (R) | 256 (R) | 32(R) | 0.38 (S) | 1024 (R) | 32 (R) | 32 (R) | 32 (R) | 256 (R) | 256 (R) | 256 (R) | 12 (R) | 0.38 (S) |
| **AF5** | 256 (R) | 256 (R) | 256 (R) | 256 (R) | 256 (R) | 32(R) | 0.5 (S) | 1024 (R) | 24 (R) | 8 (R) | 32 (R) | 256 (R) | 256 (R) | 256 (R) | 2 (R) | 0.5 (S) |
| **AF6** | 256 (R) | 64 (R) | 256 (R) | 256 (R) | 256 (R) | 32(R) | 0.38 (S) | 1024 (R) | 32 (R) | 4 (I) | 32 (R) | 256 (R) | 256 (R) | 256 (R) | 3 (R) | 0.38 (S) |
