# Supplementary material for: Genetic relatedness of serial rectal isolates of Acinetobacter baumannii in an adult intensive care unit of a tertiary hospital in Kuwait
Source: PLoS One. 2020 Apr 2;15(4):e0230976. doi: 10.1371/journal.pone.0230976 (PMC7127897; doi:10.1371/journal.pone.0230976)
Supplement: S3 Table — (DOCX) [file pone.0230976.s003.docx]

| **Patient** | **New ST** | **Gene allele for** | | | | | | |
| --- | --- | --- | --- | --- | --- | --- | --- | --- |
|  |  | ***gltA*** | ***gyrB*** | ***gdhB*** | ***recA*** | ***cpn60*** | ***gpi*** | ***rpoD*** |
| **K** | NEW 1 | 21 | 15 | 139 | 12 | 23 | * | 4 |
| **R** | NEW 2 | 1 | 87 | 3 | 62 | 2 | 110 | 3 |
| **O** | NEW 3 | 1 | 17 | 139 | 12 | ª | 170 | 5 |
| **I** | NEW 4 | 23 | 126 | 134 | 26 | 4 | 160 | 4 |
| **J** | NEW 5 | 18 | 1 | 77 | 105 | 4 | 198 | 2 |
| **J** | NEW 6 | 2 | 76 | 40 | 60 | 1 | 102 | 5 |

Table S3. Combinations of gene alleles for novel MLST.

ª new sequence for cpn60 allele

* lack of priming of the forward primer of *gpi* gene
