# Supplementary figures and images for: Genetic relatedness of serial rectal isolates of Acinetobacter baumannii in an adult intensive care unit of a tertiary hospital in Kuwait
Source: PLoS One. 2020 Apr 2;15(4):e0230976. doi: 10.1371/journal.pone.0230976 (PMC7127897; doi:10.1371/journal.pone.0230976)

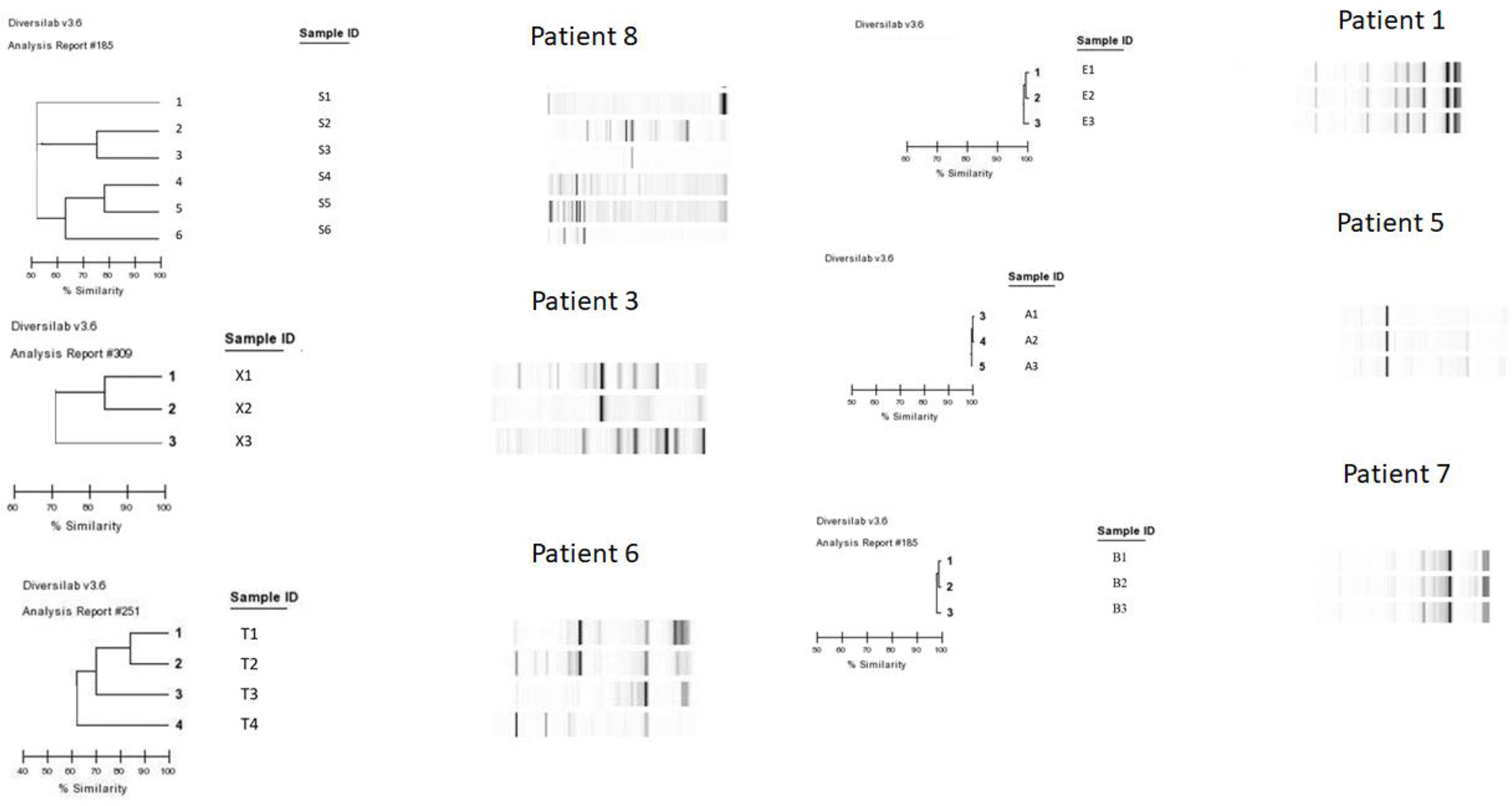

Supplement: S1 Fig — Patients 1, 5 and 7 each had similar colony morphotypes. Three colonies each from these patients were genetically identical by DiversiLab. Patients 3, 6 and 8 had 3, 4 and 6 colony morphotypes, respectively. DiversiLab analysis of these colonies showed different genetic types. (TIF) [file pone.0230976.s004.tif]

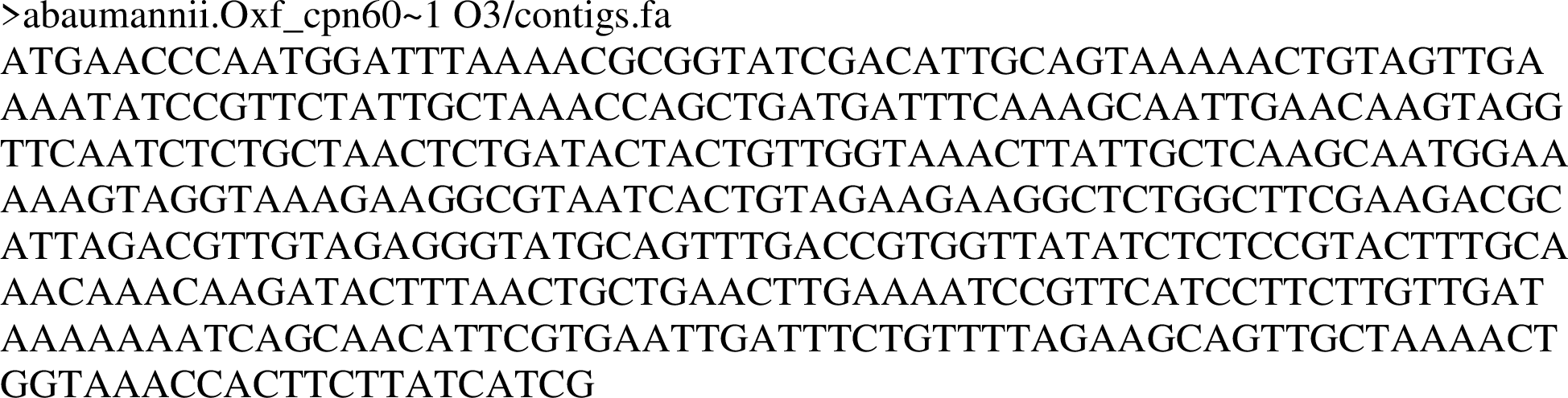

Supplement: S2 Fig — (TIF) [file pone.0230976.s005.tif]

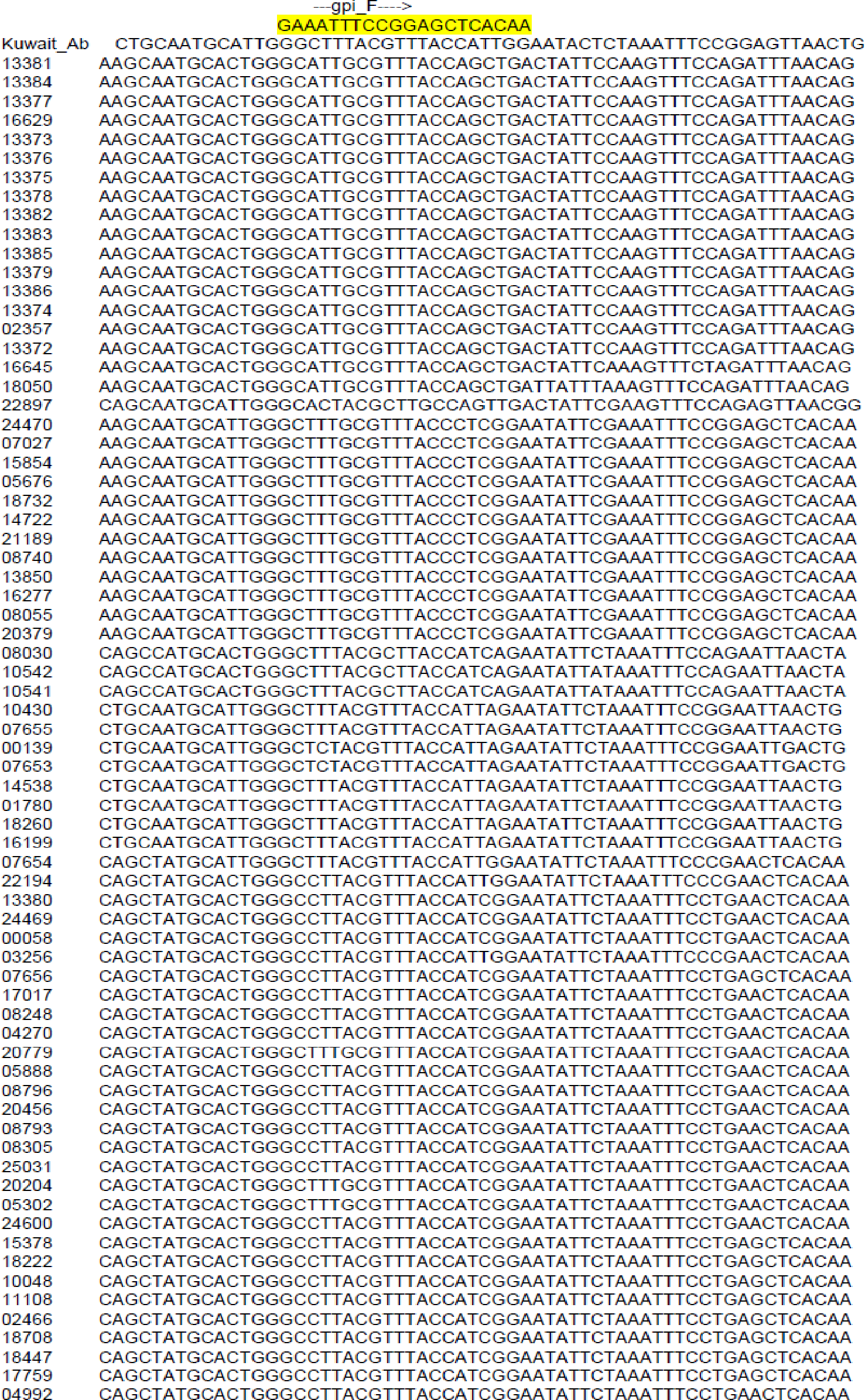

Supplement: S3 Fig — (TIF) [file pone.0230976.s006.tif]
