# Supplementary material for: Genetic relatedness of serial rectal isolates of Acinetobacter baumannii in an adult intensive care unit of a tertiary hospital in Kuwait
Source: PLoS One. 2020 Apr 2;15(4):e0230976. doi: 10.1371/journal.pone.0230976 (PMC7127897; doi:10.1371/journal.pone.0230976)
Supplement: Attachment — Submitted filename: Review_PlosOne_Ghayda_MS1.docx [file pone.0230976.s007.docx]

Al-Hashem et al. present results of a molecular surveillance study on *Acinetobacter* *baumannii* on an adult intensive care unit in a tertiary care centre in Kuwait. The study was conducted in a setting where *A. baumannii* is endemic from March 2016 to June 2017.

The surveillance was based on rectal colonization. From each specimen isolates with different morphotypes were picked and subsequently genotyped. In a pilot study the authors analyzed the association of morphotype and genotype in 12 patients. Analyses were performed with DiversiLab. Based on the pilot study data, the authors concluded that each morphotype represents one genotype.

Rectal colonization with *A. baumannii* was studied in 493 patients. In 73 out of 493 patients *A. baumannii* was detected after 72 h of admission. 32 out of theses 73 patients were positive on more than five occasions (serial isolates). Only patients with hospital-acquired (> 72 h after admission) and with serial isolates were included. The authors grouped these 32 patients in six groups based on the “colonization pattern” and picked 13 patients (2-3 from each group, 108 isolates) for further genotypic analysis (PFGE, MLST, eBURST).

The key message is the high diversity of hospital-acquired *A. baumannii* strains within one patient.

Although the authors did not use whole genome sequencing for genotyping they show with several other methods (PFGE, MLST) that patients are colonized with several different strains at single time points and during hospitalization (overtime).

The article is well-written and easy to understand. Nevertheless, there are several aspects that reduce the scientific impact of this manuscript:

Major revisions

1. The authors do not mention any antibiotic susceptibility data. I recommend adding this data as it is of interest to the reader if patients are colonized with MDR or susceptible strains.
2. The authors compared isolates from one patient and not between the patients in the genotyping analysis. From an infections control perspective it is important to know if there were any transmissions between the patients. Transmissions can be suspected if two patients hospitalized at the same time on the same ward acquired isolates with the same pulsotype/MLST-type (person-to-person- transmission). Environment-to-patient transmissions are more difficult to prove, especially retrospectively, however *A. baumannii* is known to colonize the environment. For example, PFGE patterns of isolates N3 and Y4 look similar and both patients were hospitalized during the same time period (end of 2016 and beginning of 2017). Please explain, why you decided not to compare isolates of different patients and mention it in the text (limitations?).
3. The discussion is short. Please mention more aspects (epidemiology, are there similar MLST types in the region, consequences for IPC outbreak control and typing etc.).

No limitations are mentioned. One limitation is that the sensitivity and specificity of the microbiological sampling method to detect *A. baumannii* is not known. Patients may still be colonized by the first strain overtime even if it is not detected.

4. Line 90 “*There was no outbreak during the study period.”* Please explain. What kind of outbreak do you mean? Outbreaks with any kind of bacteria or *A. baumannii*? Is there any active surveillance system in place to come to this conclusion? Please, mention in the text.
5. Isolates were considered as identical (100%), related (99-80%) or unrelated (<80%) in the PFGE analysis (Line 137-140). In my opinion, this is a very conservative approach. Even if you run the same isolate on one gel in several lanes you do not necessarily get 100% similarity. I would suggest: 100-97.5% (highly related), 97.5-80% related, <80% unrelated. You also chose a less conservative approach in the DiversLab analysis.
6. Table 1 is part of the results and not part of the methods. Table 1 shows the different groups based on colonization patterns of the patients overtime (identical, related, unrelated isolates). The six groups are complex and difficult to understand.

For example, Patient N is in group 1 “The first isolate disappears and is replaced by an identical or related isolate over time”. However, in my opinion, patient N belongs to group 3 “the first isolate disappears and is replaced by related and unrelated isolates” as there is a relatedness of “FI, -> R-> I -> R-> U”.

A better and more practical subgrouping is proposed in the discussion (line 278-280): *“colonization with identical and related isolates (patient Y), colonization with identical, related and unrelated isolates (patients N, R, S, A, AF and K), and colonization with related and unrelated isolates (patients G, J, V, O, B and I).”.*

This is the key message of the manuscript. One must consider that patients are colonized with several geno- and phenotypes over time, which is important to know in outbreak situations and to important to trace transmissions. In an outbreak you have to include several isolates from each patient in the genotyping analysis.

7. Whole genome sequencing (line 180-185): Please mention how many isolates and which isolates were analyzed by WGS and why.
8. 493 patients were screened over a period of 16 months. Please mention how many patients were excluded from the study and why (no consent?). I imagine that during the period more patients than 493 were admitted on the ICU.
9. Why did you exclude patients with *A. baumannii* present on admission (line 218)? Please explain.

Minor revisions

Line 42 – 44: Please rephrase. “*This organism has the propensity for acquiring multiple resistance genes with phenotypic expression of multidrug-resistant (MDR) characteristics. MDR strains are now endemic in many hospitals around the world, including hospitals in Kuwait [3,4] typing of the isolates to determine their relatedness.”*

Line 47 [6] Consider to mention a study where RAPD was used for *A. baumannii.*

Line 68. Please mention recent publications where WGS was used for *A. baumannii*. There are also several recent publications where a cgMLST scheme was established and used for *A. baumannii*.

Lines 117 – 118: Add %.

Line 125 “*v/v sarkosyl, pH 7.5),*” Remove the bracket.

Line 178: Please mention the website.

Line 200 – 203: Please shift to methods.

Line 245 “*These are shown Table S2*”. Please rephrase

Line 244 Please mention in the results section that the new MLST types were uploaded to the MLST server and not in the discussion section (lines 288-289).

Lines 466-474: How many isolates were included in Figure 2 and Figure 3. Please mention in the text.
