# Supplementary material for: Genetic relatedness of serial rectal isolates of Acinetobacter baumannii in an adult intensive care unit of a tertiary hospital in Kuwait
Source: PLoS One. 2020 Apr 2;15(4):e0230976. doi: 10.1371/journal.pone.0230976 (PMC7127897; doi:10.1371/journal.pone.0230976)
Supplement: Attachment — Submitted filename: Ghayda-PLOSONE-ResponseToReviewerComments.docx [file pone.0230976.s008.docx]

**Response to Reviewers**

11/2/2020

Dear Dr. Crivelleri:

Re: PONE-D-19-35790 entitled “Genetic relatedness of serial rectal isolates of Acinetobacter baumannii in an adult intensive care unit of a tertiary hospital in Kuwait”

Thank you for forwarding the reviewers’ comments and your comments. My responses are as below.

**ACADEMIC EDITOR:**

- **Dear Authors, I was in doubt for my decision, because there are many criticisms to correct.**
- **There are conflicts between the reviews, actually I think the reviewers are expressing the same concepts; the weak statistical method doesn't permit this manuscript to be accepted as it is. It needs a very accurate revision to be published.**
- **Please shorten introduction, materials and methods. Answer to all the criticism moved by the reviewers in order to make the manuscript ready for publication.**

Thank you for your decision to invite a revised manuscript in spite of many comments from the reviewers. We have made extraordinary efforts to answer the comments including shortening the Introduction and Materials & Methods.

**Reviewer #1:**

The manuscript describes the molecular typing analysis of Acinetobacter baumannii isolates from a Kuwaitian hospital. It is well written in most parts and could be interesting for the readers of PLOS One. However, due to undeniable weaknesses in methodological procedures and data interpretation, it cannot be accepted for publication in its current form.

Major comments:
**- the introduction is overlong and should be shortened. This can be easily done by a less detailed explanation of the different typing methods.**

Introduction is much shortened now (L39-L74).
**- the material and methods part is overlong and could easily be shortened by using citations (e.g. description of MLST).**

Materials and Methods section is much shortened now (L76-L157).

**- line 50: nowadays, I would not agree that PFGE is still the gold standard. Since NGS-based typing has become widespread in many countries and as this technique has a at least equivalent or in most cases higher discriminatory power, it should be named as the gold standard. However, no consensus criteria for e.g. cgMLST exist, what is the advantage of PFGE (Tenover criteria).**

This statement is modified now (L50).

**- line 52: This is not correct. PFGE has a high discriminatory power, even for isolates from different geographic regions. The real problem is, that PFGE results are not comparable from one laboratory to another due to technical variations. But one single laboratory could easily compare isolates from different regions. This has to be changed**.

This statement is changed now (L50-L52).

**- the authors did not use the Tenover criteria for outbreak investigation by PFGE. I of course know that the Tenover criteria should be used only for isolates within defined time periods, but it would be interesting to know if and how the results change when interpreted with these consensus criteria. This must be done.**

We did not investigate outbreak in our study. However, we used Tenover criteria, in addition, to interpret the relatedness of isolates (L119-L120; Table 2; L196-L197; L258-L260).

**- the band patterns shown in figure 1 are of extremely low quality for some isolates that in my opinion do not allow a reliable data interpretation. e.g. the band pattern isolate Y5a is completely unusable due to crooked bands. When looking at the bands in detail, for me it does not look like this is another pulsotype than Y5b. Additionally, the DNA amounts seem to be very different for different isolates, which complicates the analysis even more.
The same is the case for isolates R1, R3a, J11, V2 and others... The authors should reperform the PFGE for isolates with poor band patterns.**

We have repeated PFGE of these isolates several times to improve the quality of gels. What we have presented are the best patterns we have. I am afraid redoing the gels is not going to change the quality of gels or conclusions. For Y5a, even though the middle of the band is a bit drawn up, the pattern is readable. There is a misunderstanding here. We agree that Y5a and Y5b are of the same pulsotype (1c in Table 2). With regard to R1 and R3a, they are clearly different with R3a having an additional band at the top. In Table 2, these isolates are correctly labelled as 1a and 1b.

**- table 2: prior to publication, the new MLSTs and CCs must be numbered in coordination with the A. baumannii MLST website.**

Unfortunately, in spite of repeated reminders, we have not heard from the curator of the Oxford MLST scheme. Colleagues in Australia and the UK too have complained of a lack of response. Important thing is that we have presented detailed information on the novel STs. If the manuscript is accepted for publication in PLOS ONE, we will communicate the new information to the journal as and when it becomes available.
**Minor comments:
- line 44: delete "typing of the isolates to determine their relatedness" as this makes no sense here and seems to be a copy-paste issue**

Sorry for the mistake. True, it was a copy-paste issue. The sentence is deleted now (L44).
**- lines 117/118: "%" is missing for some numbers
- line 121: change it to "cultures...were typed by PFGE."**

Texts containing these lines are deleted while shortening the Introduction, and Materials & Methods.
**- line 188: "eBU_R_ST"**

The spelling mistake is corrected now (L155).

**Reviewer #2: Al-Hashem et al. present results of a molecular surveillance study on Acinetobacter baumannii on an adult intensive care unit in a tertiary care centre in Kuwait. The study was conducted in a setting where A. baumannii is endemic from March 2016 to June 2017.
The surveillance was based on rectal colonization. From each specimen isolates with different morphotypes were picked and subsequently genotyped. In a pilot study the authors analyzed the association of morphotype and genotype in 12 patients. Analyses were performed with DiversiLab. Based on the pilot study data, the authors concluded that each morphotype represents one genotype.
Rectal colonization with A. baumannii was studied in 493 patients. In 73 out of 493 patients A. baumannii was detected after 72 h of admission. 32 out of theses 73 patients were positive on more than five occasions (serial isolates). Only patients with hospital-acquired (> 72 h after admission) and with serial isolates were included. The authors grouped these 32 patients in six groups based on the “colonization pattern” and picked 13 patients (2-3 from each group, 108 isolates) for further genotypic analysis (PFGE, MLST, eBURST).
The key message is the high diversity of hospital-acquired A. baumannii strains within one patient.
Although the authors did not use whole genome sequencing for genotyping they show with several other methods (PFGE, MLST) that patients are colonized with several different strains at single time points and during hospitalization (overtime).

The article is well-written and easy to understand. Nevertheless, there are several aspects that reduce the scientific impact of this manuscript:

Major revisions

1. The authors do not mention any antibiotic susceptibility data. I recommend adding this data as it is of interest to the reader if patients are colonized with MDR or susceptible strains.**

Susceptibility data are now added (L96-L101; L185-L188).
**2. The authors compared isolates from one patient and not between the patients in the genotyping analysis. From an infections control perspective, it is important to know if there were any transmissions between the patients. Transmissions can be suspected if two patients hospitalized at the same time on the same ward acquired isolates with the same pulsotype/MLST-type (person-to-person- transmission). Environment-to-patient transmissions are more difficult to prove, especially retrospectively, however A. baumannii is known to colonize the environment. For example, PFGE patterns of isolates N3 and Y4 look similar and both patients were hospitalized during the same time period (end of 2016 and beginning of 2017). Please explain, why you decided not to compare isolates of different patients and mention it in the text (limitations?).**

This is addressed (L284-L288).
**3. The discussion is short. Please mention more aspects (epidemiology, are there similar MLST types in the region, consequences for IPC outbreak control and typing etc.).**

This is now done (L271-L280).
**No limitations are mentioned. One limitation is that the sensitivity and specificity of the microbiological sampling method to detect A. baumannii is not known. Patients may still be colonized by the first strain overtime even if it is not detected.**

Limitations are mentioned now (L281-L288).
**4. Line 90 “There was no outbreak during the study period.” Please explain. What kind of outbreak do you mean? Outbreaks with any kind of bacteria or A. baumannii? Is there any active surveillance system in place to come to this conclusion? Please, mention in the text.**

Sorry, this statement was included inadvertently. There is no active surveillance, but if there is a suspicion of outbreak due to any bacteria, then, investigation is carried out.That way, there was no separate investigation during our study period.

**5. Isolates were considered as identical (100%), related (99-80%) or unrelated (<80%) in the PFGE analysis (Line 137-140). In my opinion, this is a very conservative approach. Even if you run the same isolate on one gel in several lanes you do not necessarily get 100% similarity. I would suggest: 100-97.5% (highly related), 97.5-80% related, <80% unrelated. You also chose a less conservative approach in the DiversLab analysis.**

Because of the nature of the question asked in the study, it was necessary to adopt conservative criteria for PFGE analysis. It was a different question with regard to DiversiLab analysis. Therefore, I am afraid, the adopted approaches are justified.

**6. Table 1 is part of the results and not part of the methods. Table 1 shows the different groups based on colonization patterns of the patients overtime (identical, related, unrelated isolates). The six groups are complex and difficult to understand.
For example, Patient N is in group 1 “The first isolate disappears and is replaced by an identical or related isolate over time”. However, in my opinion, patient N belongs to group 3 “the first isolate disappears and is replaced by related and unrelated isolates” as there is a relatedness of “FI, -> R-> I -> R-> U”.
A better and more practical subgrouping is proposed in the discussion (line 278-280): “colonization with identical and related isolates (patient Y), colonization with identical, related and unrelated isolates (patients N, R, S, A, AF and K), and colonization with related and unrelated isolates (patients G, J, V, O, B and I).”.
This is the key message of the manuscript. One must consider that patients are colonized with several geno- and phenotypes over time, which is important to know in outbreak situations and to important to trace transmissions. In an outbreak you have to include several isolates from each patient in the genotyping analysis.**

We agree with this excellent suggestion and changed the grouping of patients (Table 1in Results; L199-L201).
**7. Whole genome sequencing (line 180-185): Please mention how many isolates and which isolates were analyzed by WGS and why.**

This is now mentioned (L149-L151).
**8. 493 patients were screened over a period of 16 months. Please mention how many patients were excluded from the study and why (no consent?). I imagine that during the period more patients than 493 were admitted on the ICU.**

We included all patients.
**9. Why did you exclude patients with A. baumannii present on admission (line 218)? Please explain.**

This is now explained (L180-L182).


**Minor revisions
Line 42 – 44: Please rephrase. “This organism has the propensity for acquiring multiple resistance genes with phenotypic expression of multidrug-resistant (MDR) characteristics. MDR strains are now endemic in many hospitals around the world, including hospitals in Kuwait [3,4] typing of the isolates to determine their relatedness.”**

Sorry for the mistake. The last line was inadvertently introduced by mistake while cutting and pasting. It is now deleted (L42).

**Line 47 [6] Consider to mention a study where RAPD was used for A. baumannii.**

Reference 6 is now for A. baumannii.

**Line 68. Please mention recent publications where WGS was used for A. baumannii. There are also several recent publications where a cgMLST scheme was established and used for A. baumannii.**

Now reference 18 is appropriate (L58).
**Lines 117 – 118: Add %.
Line 125 “v/v sarkosyl, pH 7.5),” Remove the bracket.**

These are deleted while shortening the Methods section.
**Line 178: Please mention the website.**

Now mentioned (L141).
**Line 200 – 203: Please shift to methods.**

Done (L104-L106)).

**Line 245 “These are shown Table S2”. Please rephrase**

Done (L166, now Table S1).

**Line 244 Please mention in the results section that the new MLST types were uploaded to the MLST server and not in the discussion section (lines 288-289).**

Done (L222-L223).

**Lines 466-474: How many isolates were included in Figure 2 and Figure 3. Please mention in the text.**

The numbers are not relevant. The figures are constructed based on sequence types.

I hope that I have answered all comments satisfactorily.

With kind regards

Yours sincerely

Professor M. John Albert

Department of Microbiology

Faculty of Medicine

Kuwait University

Kuwait
